# Ectomycorrhizal and Dark Septate Fungal Associations of Pinyon Pine Are Differentially Affected by Experimental Drought and Warming

**DOI:** 10.3389/fpls.2020.582574

**Published:** 2020-10-20

**Authors:** Catherine Gehring, Sanna Sevanto, Adair Patterson, Danielle E. M. Ulrich, Cheryl R. Kuske

**Affiliations:** ^1^Department of Biological Sciences and Center for Adaptable Western Landscapes, Northern Arizona University, Flagstaff, AZ, United States; ^2^Earth and Environmental Science Division, Los Alamos National Laboratory, Los Alamos, NM, United States; ^3^Department of Ecology, Montana State University, Bozeman, MT, United States; ^4^Bioscience Division, Los Alamos National Laboratory, Los Alamos, NM, United States

**Keywords:** climate change, dark septate endophytes, dryland ecosystems, ectomycorrhizal fungi, fungal diversity, pinyon pine, root-associated fungi, tree drought response

## Abstract

Changing climates can cause shifts in temperature and precipitation, resulting in warming and drought in some regions. Although each of these factors has been shown to detrimentally affect forest ecosystems worldwide, information on the impacts of the combined effects of warming and drought is lacking. Forest trees rely on mutualistic root-associated fungi that contribute significantly to plant health and protection against climate stresses. We used a six-year, ecosystem-scale temperature and precipitation manipulation experiment targeted to simulate the climate in 2100 in the Southwestern United States to quantify the effects of drought, warming and combined drought and warming on the root colonization (abundance), species composition and diversity of ectomycorrhizal fungi (EMF), and dark septate fungal endophytes in a widespread woodland tree, pinyon pine (*Pinus edulis* E.). Our results show that pinyon shoot growth after 6 years of these treatments was reduced more by drought than warming. The combined drought and warming treatment reduced the abundance and diversity of EMF more than either treatment alone. Individual ectomycorrhizal fungal taxa, including the drought tolerant *Cenococcum geophilum*, were present in all treatments but the combined drought and warming treatment. The combined drought and warming treatment also reduced the abundance of dark septate endophytes (DSE), but did not affect their diversity or species composition. The current year shoot growth of the trees correlated positively with ectomycorrhizal fungal diversity, highlighting the importance of diversity in mutualistic relationships to plant growth. Our results suggest that EMF may be more important than DSE to aboveground growth in *P. edulis*, but also more susceptible to the negative effects of combined climate stressors.

## Introduction

Changes in climate, including the combined effects of increased drought and warming temperatures, are significantly affecting temperate forest ecosystems (Allen C. D. et al., 2010). These stressors have already resulted in widespread tree mortality across the western United States (Breshears et al., 2005, 2009; Van Mantgem et al., 2009; Anderegg et al., 2013; Williams et al., 2013) and there is concern that significant shifts in the spatial extent and distribution of numerous tree species are imminent (e.g., Iversone and Prasad, 1998; Morin et al., 2018). However, there is also evidence that trees can acclimate to warming and drying conditions (Nicotra et al., 2010; Way and Yamori, 2014; Grossiord et al., 2017a, 2018a,b). Based on niche models, intraspecific differences among trees in morphological and physiological traits can be substantial enough to alter predictions of future plant distributions (Ikeda et al., 2017).

Microbial plant mutualists, such as root-associated fungi, significantly affect plant responses to climate change (reviewed by Kivlin et al., 2013; Mohan et al., 2014; Bennett and Classen, 2020). Many dominant temperate tree species form associations with ectomycorrhizal fungi (EMF), a diverse assemblage of ascomycete and basidiomycete fungi that improve host plant access to soil nutrients and water and provide protection from some pathogens in exchange for fixed carbon (Smith and Read, 2008). These fungi may buffer plants against climate change, but their activities and buffering ability can be affected by hot and dry conditions. Therefore, it is important to understand how root-colonizing fungi respond to environmental changes and to link those responses to the growth and survival of their plant hosts.

Ectomycorrhizal fungal responses to drought or warming have been studied in several ecosystems, but studies examining the combined effects of drought and heat stress on EMF and EMF-host plant relationships remain rare. Improvement of host plant drought tolerance by EMF has been widely documented and reviewed (Lehto and Zwiazek, 2010; Kivlin et al., 2013; Mohan et al., 2014; Gehring et al., 2017) with the strongest support for an indirect mode of action through improved host nutrition (Lehto and Zwiazek, 2010). However, drought has also been documented to lead to changes in EMF abundance, biomass, community composition and activity in pines (Karst et al., 2014). The effects of experimental warming on EMF have been less studied with an emphasis on temperate and arctic ecosystems with variable results (Mohan et al., 2014). However, temperature can be an important force structuring EMF communities, even when differences among sites in host species and associated plant communities are taken into account (Miyamoto et al., 2018; Koizumi and Nara, 2019).

The roots of many plant species, including some of those that host EMF, also are colonized by dark septate endophytes (DSE), ascomycete fungi grouped by the morphology of their highly melanized hyphae within host roots (Jumpponen and Trappe, 1998). Unlike EMF, DSE appear to lack a particular materials-exchange interface with the plant, however they may increase host plant resource uptake, particularly of organic nutrient sources (Newsham, 2011). DSE are also hypothesized to be tolerant of environmental stresses such as heat, cold, drought and salinity (Berthelot et al., 2019) and may play a role in the “fungal loop” that is thought to reduce carbon and nutrient losses in arid ecosystems by cycling them within biotic pools (Collins et al., 2008). However, there has been little research on the function of DSE in a climate change context. Kivlin et al. (2013) noted significant negative effects of inoculation with DSE on plant responses to warming in a meta-analysis but acknowledged that the results were heavily influenced by a single study of one fungal species (*Phialocephala fortinii*) and two plant species (*Picea abies* and *Betula pendula*). On the other hand, inoculation with DSE improved host plant responses to drought in the studies reviewed by Kivlin et al. (2013) and both positive and negative effects on plant biomass have been observed in more recent work on a species of arid land grass (Li et al., 2018). As with EMF, few studies have assessed the consequences of multiple climate changes on DSE-host plant relationships.

In this study, we used an ecosystem-scale field manipulation experiment to examine the consequences of drought and warming temperatures, alone and in combination, for the EMF and DSE communities associated with pinyon pine, *Pinus edulis*, a western United States tree species that occupies a large area of semi-arid landscape where it occurs with co-dominant members of the genus *Juniperus*. Warm temperatures combined with extreme drought resulted in significant *P. edulis* mortality across 12,000 km^2^ of the southwestern United States in 2002–2003 (Breshears et al., 2005). Thus, *P. edulis* has become a model for studies of the physiological basis of plant drought susceptibility (McDowell et al., 2008, 2016; Adams et al., 2009; Plaut et al., 2012; Limousin et al., 2013; Dickman et al., 2014; Sevanto et al., 2014), intraspecific variation in drought tolerance (Sthultz et al., 2009a), the biotic and abiotic legacy effects of drought induced mortality (Peltier et al., 2016; Mueller et al., 2019), and the contribution of EMF to survival and growth during drought (Gehring et al., 2014, 2017). However, the individual and combined effect of warming and drought stresses on EMF communities have not been examined and DSE have not been studied in *P. edulis*. *Pinus edulis* is often the only associate for EMF across most of its distribution in the southwestern United States (Gehring et al., 2016), while juniper and many grass and shrub species that occupy pinyon-juniper woodlands are colonized by DSE (Gehring, unpublished data).

We tested the following hypotheses: **H1**: The combined effects of drought and warming on EMF abundance, diversity and community composition will exceed the effect of either drought or heat stress alone. Warming temperatures are expected to exacerbate the effects of drought on trees in the coming years and we expect negative impacts of these combined stressors on plant symbionts. **H2**: Drought and/or warming stress will have greater negative effects on EMF than DSE because DSE are well known for their ability to tolerate stressful conditions (Berthelot et al., 2019). **H3**: Declines in EMF diversity with drought and warming will be strongly negatively associated with *P. edulis* aboveground growth. Species of EMF vary in their functional characteristics including the environmental conditions they can tolerate (Sthultz et al., 2009b; Miyamoto et al., 2018), the extent to which they colonize the soil [e.g., different hyphal exploration types (Tedersoo and Smith, 2013)], and the types of soil resources they are able to utilize (Frey, 2019). We predict that loss of EMF diversity will result in reduced functional diversity of EMFs and consequently lower plant growth because of reduced resource access capacity. We do not make a similar prediction for DSE because of their uncertain function in *P. edulis*.

## Materials and Methods

### Experimental Methods and Sampling

To examine the effects of drought and warming on EMF and DSE in *P. edulis*, roots were sampled from trees that had been under ambient (control), drought (~50% reduction in precipitation), warming (temperature 5°C above ambient) and a combination of drought and warming treatments for 6 years at the Los Alamos Survival-Mortality (SUMO) experiment located in Los Alamos County, New Mexico (35.49°N, 106.18°W, 2175 m a.s.l). The SUMO site, established in summer 2012, consists of five treatments with 5–6 trees per treatment. These treatments are: control with trees experiencing ambient temperature and precipitation, heat with trees inside open-top chambers where temperature was maintained constantly at 4.8°C above ambient temperature, drought with trees located within a precipitation exclusion structure constructed of polyethylene troughs about 1.5 m above the soil surface covering ~50% of the ground area and directing ~45% of the precipitation off the site, a combined drought and heat treatment, and a chamber control treatment with open-top chambers kept at ambient temperature (not used in this study which thus has four treatments and 20 trees total; see Pangle et al., 2012; Adams et al., 2015).

The site is located in a native pinyon-juniper woodland close to the transition zone to Ponderosa pine forest, with vegetation dominated by pinyon pine (*P. edulis* Engelm.) and one-seed juniper [*Juniperus. monosperma* (Engelm.) Sarg.], with shrubby Gambel oak (*Quercus gambelii* Nutt.) and an occasional ponderosa pine (*Pinus ponderosa* C. Lawson) occurring in the vicinity. The climate is semi-arid, with a mean annual temperature of 10.4°C (1987–2017) and a mean annual precipitation of 358 mm (1987–2017) of which about 50% falls during the North American Monsoon season from July to September (Los Alamos Weather Machine^1^). The year of our root sampling, 2018, was warmer (average temperature 12.5°C) and drier (annual precipitation 255 mm) than the 30-year average with the monsoon precipitation prior to our sampling accounting for 42% (106 mm) of the total annual precipitation, and the average temperature of June and July at the typical range of 20–21°C. The soils are Hackroy clay loam derived from volcanic tuff with a typical profile of 0–8 cm of sandy loam, 8–40 cm of clay loam and 40–150 cm bedrock. Soil depth at the site ranges from 40 to 80 cm (Soil Survey Staff, Natural Resources Conservation Service, United States Department of Agriculture^2^).

Mature *P. edulis* trees, were randomly selected for the treatments. All of the trees were >3 cm in diameter and averaged 56 ± 5 years of age based on tree cores (Grossiord et al., 2017b). The selected trees in the drought treatment were located at least 10 m from the border of the precipitation exclusion structure (equivalent to two times the height of the tallest tree in the drought treatment). In the heat treatment, the footprints of the open-top chambers ranged from 6 to 20 m^2^, and contained between one and five trees located at a minimum distance of 1.5 m from the chamber boundary and at least 5 m from any target trees in other treatments. The drought and ambient treatments form two different plots with closest target trees >80 m apart. While some root outgrowth from target trees in the combined drought and heat treatment to drought treatment or from warming treatment to ambient might have occurred, any mixing between other treatments is highly unlikely because of the distances, and most of the root system of each tree can be expected to have resided with the assigned treatment. Both *P. edulis* and *J. monosperma* were included in the experiment, and sometimes shared a chamber, but we present data only on *P. edulis* here. Previous studies conducted at this site found no differences in physiological responses between trees in the control and chamber control treatments, suggesting no indirect effect of the chambers on plant function (Adams et al., 2015; Garcia-Forner et al., 2016; Grossiord et al., 2017a,b). Therefore, we focused our sample collection only on control (*n* = 5), heat (*n* = 4), drought (*n* = 6) and combined drought and heat treatments (*n* = 5). In addition to the effects on precipitation and air temperature, the treatments influence soil temperature measured continuously at the base of all target trees with thermocouples installed at 5, 10, 15 and 30 cm depths. The drought treatment alone had negligible effect (<0.1°C) on soil temperature while the warming treatment increased soil temperature on average by 3.6°C. In April 2016, the coverage of the precipitation exclusion structure was briefly increased to 90% by adding additional clear polymer troughs to increase the stress experienced by the trees. To prevent excessive heating of the soil surface and airspace below the troughs, thermal bubble insulation was installed underneath the polymer troughs, and portable blower fans (TE-CF2421, Triangle, Jacksonville, AR, United States) were placed throughout the drought and drought and heat treatments. To ensure the effectiveness of the cooling, soil temperature was additionally measured continuously (RT-1, Decagon Devices Inc., Pullman, WA, United States) over a 0–30 cm depth at the base of each tree. Mean daily soil temperature under the structure was on average 1.4 ± 0.9°C higher than ambient conditions (see Grossiord et al., 2017a), which was clearly cooler than in the heated treatment (3.6°C above ambient). The additional precipitation exclusion was removed in April 2017, and the precipitation exclusion returned to the original ~45% coverage prior to our sampling. With this change the soil temperatures under the drought structure were similar to ambient as before.

In August of 2018, we assessed plant growth, and harvested roots from four to six pinyons from each treatment for root colonization analysis. Plant growth was determined by measuring the length of the current year shoot of ten randomly selected branches per tree using calipers. For root analyses, we collected a minimum of 200 cm fine roots (<2 mm in diameter) at a depth of 0–30 cm, pooled from two locations per tree. Roots were collected right at the tree base and well within each treatment footprint, traced to the focal tree, carefully excavated using a trowel, and placed in a cooler prior to transport to Northern Arizona University where they were stored at −20°C until processing. Root colonization by EMF was measured on each sample by counting the number of living ectomycorrhizal root tips relative to non-colonized root tips based on differences in their morphology as described in Gehring and Whitham (1991). Living ectomycorrhizal root tips (~75/tree) were then removed and examined under a dissecting microscope at 20X magnification to categorize them morphologically based on color, texture, hyphal quantity and structure (Agerer, 1991). Hyphal exploration type was assessed by observing each morphotype for emanating hyphae and presence of rhizomorphs (Agerer, 1991; Tedersoo and Smith, 2013), in addition to utilizing the Agerer (2006) categorization of EMF genera. Two morphotypes had not been observed in previous studies of *P. edulis* in the Gehring lab and were hand sectioned to look for a Hartig net, the specialized exchange structure characteristic of EMF (Smith and Read, 2008). Root tips were stored in separate tubes by morpohotype/tree at −20°C until molecular analysis of fungal communities.

To assess DSE colonization, a sample of the remaining fine roots from each sample (~50 cm, lacking EMF colonized root tips) was cleared for 20 min in boiling 10% KOH and then left an additional 12 h at room temperature in fresh 10% KOH followed by several rinses in tapwater. Around 10-1 cm segments of root were mounted on glass slides, and observed using a compound microscope at 400× magnification. The presence of melanized, septate hyphae and microsclerotia were used as indicators of DSE and quantified using the grid-line intersect method (McGonigle et al., 1990) using ~100 intersections per sample. Root samples were not stained as melanized hyphae were clearly visible without this step as observed in other study systems (Liu et al., 2017; Hughes et al., 2020). The remaining fine roots were stored at −20°C until molecular analysis of fungal communities.

### Molecular Characterization of Fungal Communities

Standard methods for DNA extraction, PCR, and Sanger sequencing for EMF root tips were used (e.g., Gehring et al., 2017; Patterson et al., 2018). Briefly, we extracted DNA from one to five root tips (depending on availability) of every fungal morphotype found on every tree using the High Molecular Weight DNA Extraction protocol of Mayjonade et al. (2016). We performed polymerase chain reaction (PCR) under conditions described by White et al. (1990) and Gardes and Bruns (1993), to amplify the internal transcribed spacer (ITS) region of the rRNA of the fungal genome with the ITS1-F (CTTGGTCATTTAGAGGAAGTAA) and ITS4 (TCCTCCGCTTATTGATATGC) primer pair as in White et al. (1990) and Gardes and Bruns (1993), using KAPA Taq Hotstart (Kapa Biosystems, Wilmington, MA 01887, United States). Successfully amplified PCR product was purified and then cycle sequenced using BigDye Terminator Mix 3.1 (Thermo Fisher Scientific Inc.). Sequencing was performed on an ABI 3730xl Genetic Analyzer (Applied Biosystems, Foster City, California, United States) at the Environmental Genetics and Genomics Laboratory at Northern Arizona University. When amplification or sequencing of a morphotype was unsuccessful, an additional root tip from that morphotype from that tree was processed.

We sequenced the fine roots described above to assess DSE community characteristics using the Illumina platform. We extracted DNA from 2.0 g wet mass samples (one per tree) using DNeasy Plant Extraction Kits (Qiagen, Valencia, CA, United States). PCR was performed using primers and conditions described by Taylor et al. (2016) to amplify the ITS region of the rRNA of the fungal genome with the ITS4-FUN and 5.8S-FUN primer pair (Taylor et al., 2016) using Phusion High-Fidelity DNA Polymerase (New England Biolabs, Ipswich, MA, United States). PCR products were checked on a 1% agarose gel, pooled, diluted 10-fold, and used as the template in the subsequent tailing reaction with region-specific primers including the Illumina flow cell adapter sequences and an eight-nucleotide barcode. Products of the tailing reaction were purified with carboxylated SeraMag Speed Beads (Sigma-Aldrich, St. Louis, Missouri, United States) at a 1:1 v/v ratio as described in (Rohland and Reich, 2012), and quantified by PicoGreen fluorescence. Equal quantities of the reaction products were then pooled. The library was bead-purified once again (1:1 ratio), quantified by qPCR using the Library Quantification Kit for Illumina (Kapa Biosciences, Woburn, Massachussetts, United States), and loaded at 9 pM (including a 30% PhiX control) onto an Illumina MiSeq instrument (Illumina, San Diego, California, United States) using 2 × 150 paired-end read chemistry.

### Data Analysis

DNA sequences of EMF root tips were aligned and trimmed in Bioedit (Hall, 1999) and identified to the genus or species level using the Basic Logical Alignment Search Tool (BLAST; Altschul et al., 1990) and UNITE (Kõljalg et al., 2013) databases. We considered sequence similarity of ≥98% to published sequences indicative of species-level identity and 95–97% indicative of genus-level identity (Kõljalg et al., 2013).

For the DSE data set, the forward and reverse reads of ITS sequences were stitched using FastqJoin (Aronesty, 2011) and quality filtered using the software package Quantitative Insights into Microbial Ecology v 1.9 (QIIME; Caporaso et al., 2010) using a Phred score cut-off value of 20. DNA sequences were extracted using ITSx (Bengtsson-Palme et al., 2013), and OTUs were picked using SWARM (Mahé et al., 2014) with a local clustering threshold value of 3. The most abundant sequence for each operational taxonomic unit (OTU) was aligned with PyNAST (Caporaso et al., 2010) against the UNITE (ITS; Nilsson et al., 2019) database using a 97% similarity cutoff, and taxonomy was assigned using BLAST (Altschul et al., 1990). Community composition data generated from amplicon counts were CSS-normalized and OTU tables were filtered to putative DSE taxa including the following orders: Helotiales, Xylariales, Pleosporales, Sordariales, Hypocreales and Chaetosphaeriales (Grünig et al., 2008).

Community composition of EMF and DSE was compared among treatments using separate Permutational MANOVAs (PERMANOVA) with the Bray-Curtis dissimilarity index in Primer 7 (Primer-e Ltd., Ivybridge, United Kingdom). The Shannon diversity (H′ log base e) was calculated for EMF and DSE using Primer 7 and compared among treatments using a one-way ANOVA in SPSS (IBM SPSS v. 20) followed by a Tukey’s test to locate treatment differences. Data on EMF colonization, DSE colonization, and shoot growth also were analyzed using one-way ANOVAs followed by Tukey’s tests. Hyphal exploration type was evaluated using a MANOVA in SPSS.

## Results

### EMF Colonization and Community Composition

**Figure 1:**
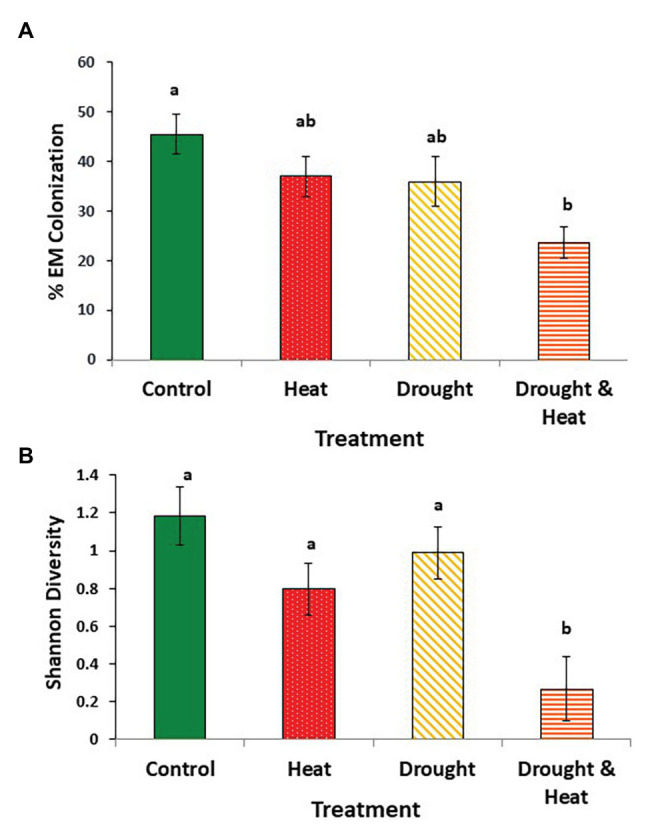
Mean (+/− 1 S.E.) ectomycorrhizal fungal (EMF) colonization **(A)**, and Shannon diversity index of the EMF communities **(B)** found in the roots of pinyon pine trees grown under ambient (control), warming (+4.8°C compared to ambient), drought (−50% of precipitation) and combined heat and drought treatments for 6 years. Different letters above the bars denote differences among groups at *p* < 0.05.

Root colonization by EMF was, on average, 50% lower in trees that experienced both drought and warming than in trees that experienced ambient conditions (*F*_3,16_ = 3.573, *p* = 0.038; Figure 1A). Colonization by EMF was intermediate in the drought only or warming only treatments (Figure 1A). Similar patterns were observed with Shannon diversity which was, on average, 4.4X greater on control trees than trees in the combined drought and warming treatment (*F*_3,16_ = 4.389, *p* = 0.02; Figure 1B). Again, trees that experienced only drought or warming were intermediate, but closer to the ambient treatment than to the combined treatment (Figure 1B).

**Figure 2:**
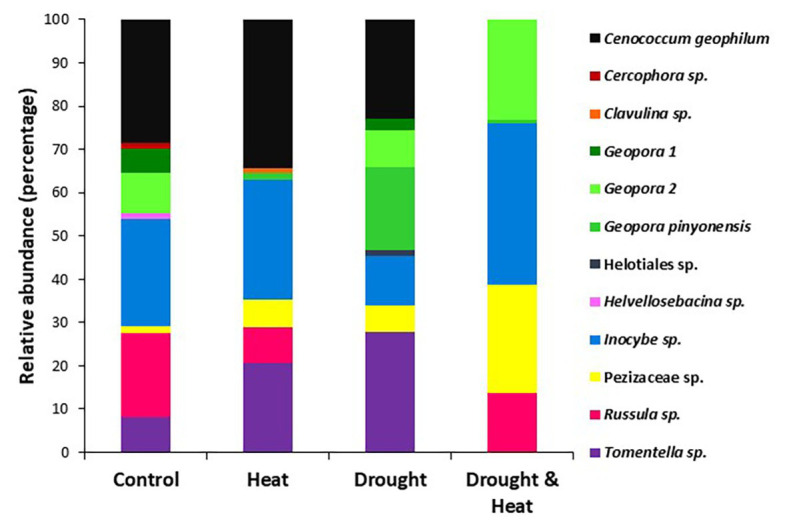
Relative abundance of the EMF species found in the roots of pinyon pine trees grown under ambient (control), heat (+4.8°C compared to ambient), drought (−50% of precipitation) and combined heat and drought treatments for 6 years. The species in control and heat treatments resembled each other, while significantly fewer species were found in the combined drought and heat treatment.

The root tip EMF community consisted of 12 species, seven members of the Phylum Ascomycota and five members of the phylum Basidiomycota (Table 1; Figure 2). Low species richness and dominance by fungi in the Ascomycota is typical of *P. edulis* (Gehring et al., 2014; Patterson et al., 2018). The two members of the Ascomycota not observed in previous studies of *P. edulis* (e.g., Patterson et al., 2018; Mueller et al., 2019), *Cercophora* sp. and Helotiales sp. produced consistent morphotypes with obvious fungal mantles, but microscopy indicated poorly formed Hartig nets. These fungi were rare. They were observed in only one treatment each where they made up less than 2% of the community, but they were included in subsequent statistical analyses despite the poorly formed Hartig net. Recent research indicates that EM fungi can still carry out critical functions even without a functional Hartig net (Sa et al., 2019). Members of the Heliotiales can form associations with ectomycorrhizas (Nakamura et al., 2018) and it is possible that this is what we observed.

**Table 1:** Ectomycorrhizal fungal taxa identified on *Pinus edulis* using ITS sequences.

ID	Fungal phylum^1^	Hyphal exploration type^2^	Matching GenBank accession number^3^	Query coverage%^4^	Identity%^5^	GenBank accession number^6^
*Cenococcum geophilum*	A	Short	MK131420.1	100	99	n/a
***Cercophora* sp**.	A	Short	KX171944.1	95	96	MW026419
***Clavulina* sp**.	B	Short	MK627472.1	88	99	MW026416
*Geopora pinyonensis*	A	Short	KF546493.1	99	99	n/a
*Geopora* 1	A	Short	KF546490.1	98	99	n/a
*Geopora* 2	A	Short	KF546492.1	98	99	n/a
*Helotiales* sp.	A	Short	HM488537.1	99	99	n/a
***Helvellosebacina* sp.**	B	Short	KF000456.1	96	97	MW026417
***Inocybe* sp.**	B	Short	MG833870.1	96	97	MW026420
***Pezizaceae* sp.**	A	Short	AJ633598.1	100	97	MW026421
***Russula* sp.**	B	Contact	KM402893.1	97	98	MW026415
***Tomentella* sp.**	B	Short	EU444541.1	92	98	MW026418

1 Indicates Ascomycota (A) or Basidiomycota (B).

2 Hyphal exploration type based on our observational measurements, Agerer (2006) and Tedersoo and Smith (2013).

3 Accession number in NCBI GenBank (https://www.ncbi.nlm.nih.gov/genbank/) that most closely matches the sequences generated in this study.

4 Query coverage indicates the percentage of the query sequence that overlaps the reference sequence.

5 Identity percent indicates the similarity of the query sequence and the reference sequence across the length of the coverage area.

6 DNA sequences of taxa in bold type did not match GenBank sequences at >99% identity with >95% query coverage. These sequences were submitted to GenBank and their accession numbers provided.

While overall EMF community composition was similar among treatments (pseudo *F*_3,19_ = 1.35, *p* = 0.163), individual taxa were significantly affected by the combined drought and warming treatment (Figure 2). The relative abundance of both *Cenococcum* sp. and *Tomentella* sp. varied among treatments owing to their absence from the combined drought and warming treatment (*Cenococcum* sp. pseudo *F*_3,19_ = 1.72, *p* = 0.021, *Tomentella* sp. pseudo *F*_3,19_ = 1.97, *p* = 0.005; Figure 2). Only contact and short hyphal exploration types were observed, with short exploration type dominating in all treatments [mean (S.E.) % short exploration type for control trees = 79.3 (10.48) for drought only trees = 100 (0.0), for heat only trees = 90.6 (8.04) and for drought and heat combined = 86.3 (13.6); *F*_3,16_ = 0.982, *p* = 0.462].

### DSE Colonization and Community Composition

**Figure 3:**
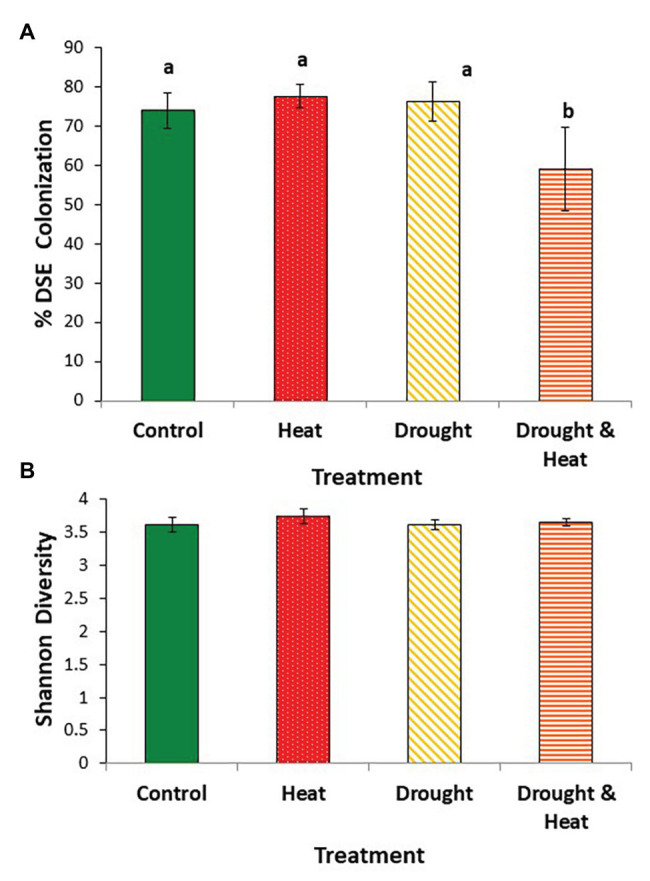
Mean (+/− 1 S.E.) root colonization by dark septate endophytes (DSE) **(A)**, and Shannon diversity index of the DSE communities **(B)** found in the roots of pinyon pine trees grown under ambient (control), warming (+4.8°C compared to ambient), drought (−50% of precipitation) and combined heat and drought treatments for 6 years. Different letters above the bars denote differences among groups at *p* < 0.05.

As with EMF, root colonization by DSE was negatively affected by the combined drought and warming treatment. Colonization by DSE was high (~75%, on average) in the ambient, drought and warming treatments, but was ~20% lower in the combined drought and warming treatment (*F*_3,16_ = 4.532, *p* = 0.018; Figure 3A).

The root DSE community consisted of 101 OTUs, with most of these (57%) occurring at less than 1% relative abundance in any treatment group. Thirty-two percent of the OTUs were identified to species, 31% to genus, 9% to family, 20% to order, and 8% to phylum (Ascomycota). The genus *Cladophialophora* had the most OTUs (*n* = 11) followed by *Paraphoma* (*n* = 6), while the most common OTUs identified at the ordinal level were found in the Pleosporales and Helotiales, with six OTUs each.

**Figure 4:**
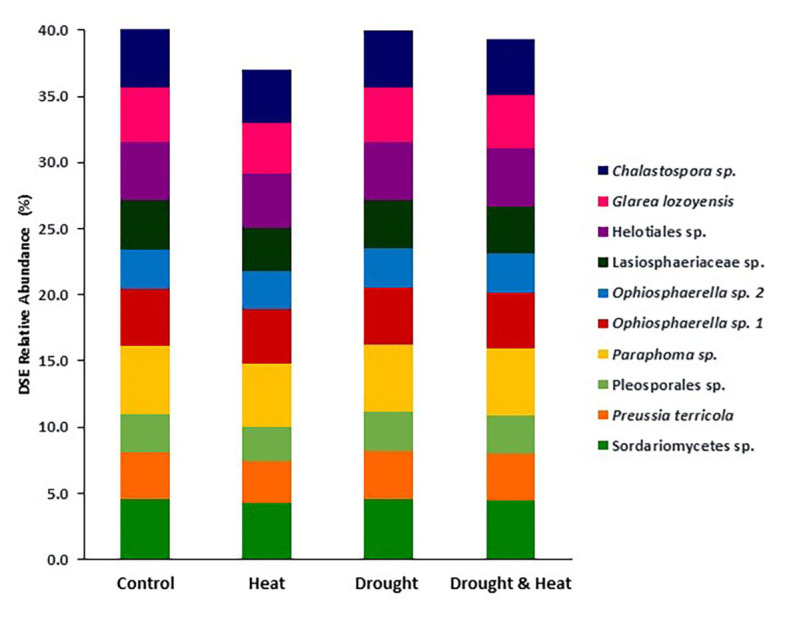
Relative abundance of the 10 most abundant taxa of DSE found in the roots of pinyon pine trees grown under ambient (control), heat (+4.8°C compared to ambient), drought (−50% of precipitation) and combined heat and drought treatments for 6 years. There were no significant differences in DSE community composition among treatments.

In contrast to observations with EMF, Shannon diversity at the OTU level was similar in all four treatments (*F*_3,16_ = 0.397, *p* = 0.757; Figure 3B). DSE community composition was also similar among groups (pseudo *F*_3,16_ = 1.35, *p* = 0.163). This similarity is illustrated by the relative abundance of the 10 most common OTUs which make up between 36 and 40% of the community in all four treatments (Figure 4).

### Shoot Growth and Relationships to Fungal Colonization and Diversity

**Figure 5:**
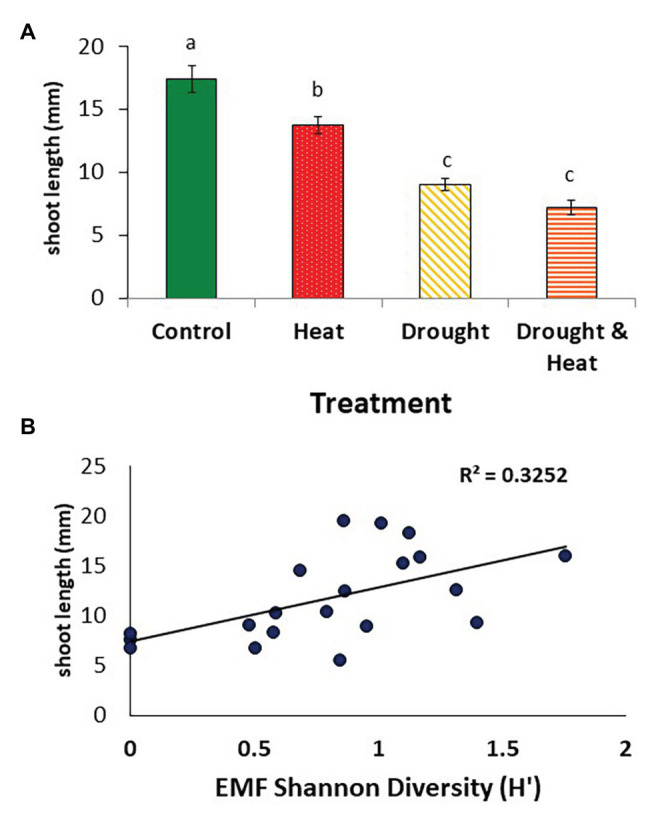
Mean (+/− 1 S.E.) length of the current year shoots observed in pinyon pine trees in the year of root collection after growing under the control, heat, drought, and combined drought and heat treatment for 6 years **(A)**. The length of the current year shoots in these trees correlated positively with the Shannon diversity of the root ectomycorrhizal fungal communities **(B)**.

Pinyons growing in ambient conditions had the greatest mean shoot elongation during the year fungi were sampled, followed by the warming only treatment (*F*_3,16_ = 40.325, *p* < 0.001; Figure 5A). Pinyons experiencing drought only or drought and warming had similar mean shoot lengths, which were approximately 50% lower than those of pinyons in the ambient treatment and approximately 40% lower than pinyons in the warming only treatment (Figure 5A).

Mean shoot length during the growing season in which fungi were sampled was most strongly positively correlated with EMF diversity (*R*^2^ = 0.3252, *F*_1,18_ = 7.022, *p* < 0.01, Figure 5B), but also positively correlated with EMF colonization (*R*^2^ = 0.224, *F*_1,18_ = 5.128, *p* = 0.035). There was no association between shoot growth and DSE colonization (*R*^2^ = 0.07, *F*_1,18_ = 1.486, *p* = 0.239) or DSE diversity (*R*^2^ = 0.006, *F*_1,18_ = 0.104, *p* = 0.751, data not shown).

## Discussion

### Declines in Ectomycorrhizal Fungal Colonization and Diversity

Consistent with our first hypothesis, root colonization by EMF was most negatively affected in the combined drought and warming treatment, but also reduced in the drought or warming only treatments. Drought alone has been shown to cause reductions in EM colonization in many of the studies reviewed by Mohan et al. (2014) and Karst et al. (2014) and in a more recent study of beech (*Fagus sylvatica*; Köhler et al., 2018). The main effect of the drought treatment we implemented was to reduce precipitation reaching the ground by ~45%. This reduced the capacity of small precipitation events to replenish soil moisture reserves so that the relative water content extractable by plants at the top 30 cm of the soil remained on average ~50% lower compared to control and heat treatments throughout the experiment. It did not change the absolute maximum soil moisture content measured during snow melt or after the heaviest monsoon rains, or the minimum soil moisture content measured in the end of the dry season each year. These precipitation events and drought periods were strong enough to drive all the treatments to similar soil moisture content. But, during less extreme precipitation seasons, drought treatment caused plant extractable soil moisture content to fluctuate around 20% in the drought treatments compared to 40% in control and heat treatments (Grossiord et al., 2017a,b) significantly reducing water availability in the soil.

Our findings regarding warming temperatures are difficult to compare to other research as previous field studies have focused on arctic or boreal ecosystems where warming temperatures frequently led to increased EM colonization (Mohan et al., 2014; Bennett and Classen, 2020). At our site, the soil temperatures at 10 cm depth (the average depth from which roots were collected) can reach up to 60°C even without additional heating, exceeding the environmental tolerance of many species of fungi (Maheshwari et al., 2000). Experimental heating increased temperatures in our study system an average of 4.8°C (Adams et al., 2015; Garcia-Forner et al., 2016) which increased the peak temperatures proportionally and reduced the time spent at below freezing temperatures in the winter by roughly 50% compared to the ambient and drought treatments. The large reduction in EM colonization in the combined heat and drought treatment suggests that sustained warm temperatures or heat waves during periods of drought may limit the ectomycorrhizal symbiosis in semi-arid environments. A critical question that remains is if the reductions in EM colonization we observed also limit EMF propagule production and viability, and thus has a lasting effect on the inoculum potential of the soil.

In addition to a sharp decline in the abundance of EMF in the combined heat and drought treatment, EMF diversity dropped by more than 75% relative to the ambient control. Previous studies have documented that EMF diversity declines with drought (Karst et al., 2014; Mohan et al., 2014) while studies of warming temperatures have again focused largely on arctic or boreal systems where results have been mixed (Mohan et al., 2014; Fernandez et al., 2017). In pinyon pine, long-term drought resulted in reduced EMF diversity (Sthultz et al., 2009b; Gehring et al., 2014). Restoring moister conditions to pinyon pines in the same study area with experimental watering during drought did not increase EMF diversity, suggesting that reductions in diversity with drought may be long term (Patterson et al., 2018). While warming experiments (Fernandez et al., 2017) and drought (Gehring et al., 2014; Karst et al., 2014) appear to favor members of the Ascomycota, their dominance did not differ among treatments in our study. In fact, one of the more drought tolerant species of fungi, the ascomycete, *Cenococcum geophilum* (Pigott, 1982; Jany et al., 2003), was common (average 31% relative abundance) in the control, heat and drought treatments, but absent from the combined heat and drought treatment (Figure 2). However, members of the genus *Geopora*, documented to promote drought tolerance in *P. edulis* (Gehring et al., 2017) had their highest abundance in the drought only treatment but also were present (~25% relative abundance) in the combined heat and drought treatment. Members of this genus are found in numerous stressful environments including mine spoils (Hrynkiewicz et al., 2009) and post-fire landscapes (Fujimura et al., 2004) but the mechanisms contributing to their success in these challenging environments are unknown. Studies in cooler, wetter ecosystems, have reported that warming increased EMF taxa with presumably less energetically expensive short distance hyphal exploration types (Fernandez et al., 2017), but EMF taxa with short exploration types dominated in all treatments in our study, consistent with previous studies of *P. edulis* (Patterson et al., 2018).

### Small Effect of Treatments on DSE

Colonization by DSE was high in all groups, exceeding 50%, and only declined slightly in the combined drought and warming treatment. DSE diversity and species composition was unaffected by any of the temperature and precipitation reduction treatments. This lack of change relative to the large reductions in diversity and colonization observed in EMF is consistent with our second hypothesis. DSE are well known for their high abundance in stressful environments, including arid lands (Porras-Alfaro et al., 2008; Porras-Alfaro and Bayman, 2011), and were previously observed to be less responsive to changes in the abiotic environment than mycorrhizal fungi (Bueno de Mesquita et al., 2018). DSE may have been affected to a lesser extent than EMF because of the stress tolerance of their highly melanized hyphae. One function of melanin in fungi is protection from harmful environmental conditions including ultraviolet radiation and temperature extremes (Butler and Day, 1998). Interestingly, *C. geophilum*, the EMF taxon that was common in all treatments but the heat and drought treatment is also heavily melanized. Melanin inhibition studies on *C. geophilum* isolates showed that fungal growth was negatively affected only when isolates were subjected to osmotic and desiccation stress (Fernandez and Koide, 2013). Comparative studies of DSE and EMF like *C. geophilum* would be helpful to elucidate the importance of melanin to their stress tolerance and that of their host plants.

Although we observed DSE taxa commonly found in members of the Pinaceae like *Phialocephala fortinii* (Jumpponen and Trappe, 1998; Grünig et al., 2008), these taxa were less abundant than members of the genera *Chalastospora* and *Paraphoma* that are better known as plant pathogens than endophytes. One of the most common genera we observed, *Paraphoma*, made up ~5% of all sequences across treatments but we could not find reference to its occurrence in members of the Pinaceae. *Paraphoma* can cause root rot in crops such as alfalfa resulting in necrotic lesions (Cao et al., 2020). However, we did not observe damage to the roots we sequenced or observed microscopically. Our results highlight how much remains to be learned about DSE. Their high taxonomic diversity within the Ascomycota and function along the mutualism-parasitism axis are well documented (Berthelot et al., 2019), but also present challenges to understanding their influence on host plant growth and survival.

### Fungal Relationships to Host Plant Growth

We observed significant associations between *P. edulis* aboveground growth and the abundance and diversity of EMF but not DSE, consistent with our third hypothesis. Similar to previous observations at our site (Grossiord et al., 2017b), current year shoot growth was reduced relative to controls in the drought and combined drought and heat treatment, but not in the heat treatment alone (Figure 5). Although the drought treatment slightly negatively affected EMF colonization but not diversity, there was a higher correlation between EMF diversity and growth than EMF colonization and growth with EMF diversity explaining 32.5% of the variation. In a study of the EMF communities of *P. edulis* that remained following host plant mortality, reduced diversity due to the absence of EMF in the genus *Tuber* was associated with reduced seedling size (Mueller et al., 2019). While our results suggest that EMF may be more important to aboveground growth than DSE, we did not measure belowground growth. DSE may have influenced pinyon root length or biomass, important contributors to the beneficial effects of DSE in other arid land plant species (Li et al., 2018).

Few studies have experimentally manipulated EMF diversity to understand mechanisms with mixed results (Baxter and Dighton, 2001; Jonsson et al., 2001; Hazard et al., 2017). Studies are even more limited in low moisture, high temperature environments, but phosphorus uptake efficiency was observed to decrease due to reductions in EMF diversity under low soil moisture conditions in European beech (Köhler et al., 2018). At our site, plant phosphorus uptake has not been studied, but warming increased both nitrification processes and the amount of nitrate in the root zone, while drought increased the amount of ammonium, and both these effects were present in the combined heat and drought treatment (Grossiord et al., 2018a). These shifts did not have any effect on the N content of plant tissues, plant N allocation or preference for using nitrite or ammonium suggesting that nitrogen was not limiting growth. But, these changes in N dynamics could contribute to the composition and function of the root-zone microbiome given the importance of N to EMF communities in many other ecosystems (Lilleskov et al., 2019). Nitrogen fertilization increased leaf production and reduced EMF abundance in *P. edulis* (Allen M. F. et al., 2010). However, it also was associated with increased tree mortality in *P. edulis* during drought, possibly due to a reduced role of EMF in water uptake (Allen M. F. et al., 2010). Thus changes in N dynamics in this study system could become significant to plant growth and vitality as drought and warming continue. In beech (*Fagus sylvatica* L.), moderate drought increased the importance of EMF to uptake of inorganic N, but this effect was EMF species specific, even differing among members of the same genus (Pena and Polle, 2014). *C. geophilum*, the taxon shared between our study and that of Pena and Polle (2014), did not improve N uptake under drought.

In our study, we cannot determine conclusively if changes in fungi influenced host plants or the reverse (or a combination), but previous studies utilizing the same experiment provide clues. Over the years, the drought, heat and combined drought and heat treatments have affected the carbon fixation and water uptake as well as growth of the *P. edulis* trees. Drought and combined drought and heat treatments have shown significantly lower average stomatal conductance and photosynthesis at saturation light (Grossiord et al., 2017a, 2018a). These changes were combined with delayed initiation of both shoot (Adams et al., 2015; Grossiord et al., 2017b) and stem growth (Manrique-Alba et al., 2018) in the combined heat and drought treatment, reduced needle elongation in both the drought and combined heat and drought treatments (Grossiord et al., 2017b), and reduced capacity to replenish stem water reserves in the combined drought and heat treatment (Grossiord et al., 2017c; Manrique-Alba et al., 2018). These observations suggest reduced plant productivity that could influence the ability to attract and maintain mutualistic fungi. While EMF can access nutrients in soil organic matter through a variety of mechanisms (Frey, 2019) they generally rely on photosynthates from their hosts, but potentially to varying degrees (Koide et al., 2008). For example, species richness in EMF associated with European beech trees was affected by stem girdling that reduced direct transport of photosynthates to the roots (Pena et al., 2010). In addition to the protection provided by melanin for DSE, our observed differences in drought and heat effects on EMF and DSE colonization and species richness could be explained by different degree of fungal dependency on plant-produced carbohydrates between these groups.

At our site, reductions in plant photosynthesis and growth were accompanied by reduction in leaf-area-specific plant hydraulic conductivity, but no change in the depth of main water sources used by the trees (Grossiord et al., 2017a), or the leaf area: sapwood area ratio (McBranch et al., 2019). These findings suggest that the trees adjusted their water demand to water availability without changing anatomical structure or rooting depth, even if the heat and drought treatment increased competition for water in the main water source layer by inducing a shift that brought the main water source for co-occurring grasses to the same layer (Grossiord et al., 2019). Whether this shift affected DSE communities differently from EMF communities is unclear, nor do we understand how the two groups of fungi interact within roots or soil. There is evidence that DSE colonization has positive effects on AMF colonization of grass roots in arid grasslands (Menoyo et al., 2020), while interactions between EMF and DSE appear to be species and strain specific (Berthelot et al., 2019). In most pinyon-juniper woodlands, DSE have multiple hosts, while EMF are restricted to *P. edulis*; this difference also may contribute to the different sensitivities of the two groups to the combined stressors in our study.

## Conclusion

Our experimental study of the effects of warming and drought on the fungal communities of an arid land conifer provides an important contrast to similar studies in cooler, wetter climates. Heating alone caused little change, but combined heat and drought had strong negative effects on root-associated fungi. Our results also indicate that EMF are more sensitive than DSE, with the former showing declines in both abundance and diversity. The differences among root symbionts could be due to differences in stress tolerance, host plant specificity, degree of dependence on plant hosts for carbon, or a combination of these factors. Our data on aboveground plant growth and EMF species diversity support the view that EMF are mutualists, and emphasizes the importance of community diversity rather than simple abundance to plant vitality. Less is known about the importance of DSE to plant performance in arid land trees or how DSE and EMF interact with one another and thereby affect their shared host. Obtaining this information is critical for understanding potential acclimation and adaptation of forest ecosystems to changing climate as well as for predicting bottle necks and tipping points that influence forest health.

## Data Availability Statement

The raw data supporting the conclusions of this article will be made available by the authors, without undue reservation.

## Author Contributions

CG contributed to data collection and analysis and led the writing of the manuscript. SS helped to construct and maintain the experiment, contributed to data collection, and helped to draft the manuscript. AP and DU contributed to data collection and revised the manuscript. CK initiated the collaboration and revised the manuscript. All authors contributed to the article and approved the submitted version.

### Conflict of Interest

The authors declare that the research was conducted in the absence of any commercial or financial relationships that could be construed as a potential conflict of interest.

## Footnotes

^1^https://weathermachine.lanl.gov/

^2^http://websoilsurvey.nrcs.usda.gov
